# Effects of Schizonepetin on Activity and mRNA Expression of Cytochrome P450 Enzymes in Rats

**DOI:** 10.3390/ijms131217006

**Published:** 2012-12-12

**Authors:** Beihua Bao, Ting Geng, Yudan Cao, Weifeng Yao, Li Zhang, Anwei Ding

**Affiliations:** 1 Jiangsu Key Laboratory for High Technology Research of TCM Formulae, College of Pharmacy, Nanjing University of Chinese Medicine, 138 Xianlin Rd, Nanjing 210046, China; E-Mails: beihua.bao@gmail.com (B.B.); raindc@163.com (Y.C.); weifengyao@sohu.com (W.Y.); zhangliguanxiong@163.com (L.Z.); 2 Jiangsu Kanion Pharmaceutical Co., Ltd., 58 Haichang South Road, Xinpu District, Lianyungang 222001, China; E-Mail: yilinger110@126.com

**Keywords:** Schizonepetin, Cytochrome P450, substrates drugs, pharmacokinetic parameters, mRNA, real-time RT-PCR

## Abstract

The aim of this study was to find out whether Schizonepetin influences the pharmacokinetics of the main substrates drugs of CYP1A2, CYP3A1/2, CYP2E1, CYP2C19 and CYP2D6 in rats; the influence on the levels of CYP mRNA was also studied. Phenacetin, dapsone, chlorzoxazone, omeprazole and metoprolol were selected as probe substrates for CYP1A2, CYP3A1/2, CYP2E1, CYP2C19 and CYP2D6 respectively. HPLC methods were employed for the determination of these substrates in plasma and the pharmacokinetic parameters were calculated. Real-time RT-PCR was used to determine the effects of Schizonepetin on the mRNA expression of CYP3A1, CYP1A2 and CYP2E1 in the rat liver. After the rats were orally administrated with Schizonepetin once a day for seven consecutive days, there were significant differences in plasma concentration of phenacetin, dapsone, chlorzoxazone and metoprolol, but not omeprazole, as compared with pre-administration. In addition, Schizonepetin induced the expression of CYP3A1, CYP1A and CYP2E1 at dosages of 24 and 48 mg/kg. Our results indicated that Schizonepetin had significant induction effects on CYP3A1/2 and inhibition effects on CYP1A2, CYP2E1 or CYP2D6 as oriented from the pharmacokinetic profiles of the substrates. Moreover, in the mRNA expression levels, Schizonepetin could induce the mRNA expression of CYP3A1, CYP1A and CYP2E1. In conclusion, co-administration of some CYP substrates with Schizonepetin may lead to an undesirable herb-drug interaction.

## 1. Introduction

**Figure 1:**
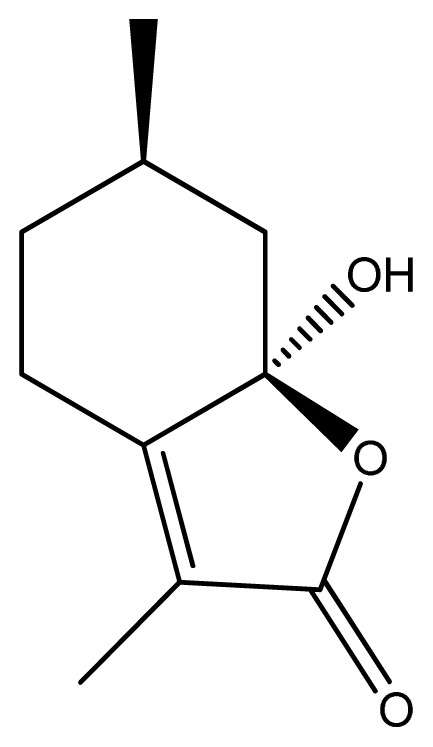
Chemical structure of Schizonepetin.

Herba *Schizonepetae* (“Jingjie” in Chinese) originated from dried aerial parts of *Schizonepeta tenuifolia* Briq. has been frequently and widely used in the treatment of colds and high fevers in China for centuries [1]. The essential oil was considered responsible for most of the therapeutic benefits of Herba *Schizonepetae*. Schizonepetin (Figure 1), a natural monoterpene, was the main component and was first isolated from the essential oil of *S. tenuifolia* in our laboratory [2]. It possessed various pharmacological effects, including excellent antiviral, anti-inflammatory and analgesic activities [2,3], while without obvious toxicity of the respiratory, cardiovascular and nervous systems in rats and dogs [4].

Previously, the pharmacokinetic profile of Schizonepetin after oral and intravenous administrations in rats has been studied in our laboratory. Schizonepetin could be fast and well absorbed as its *C*_max_ and absolute bioavailability were 0.80 h and 75%, respectively. In acute and subacute toxicity studies, it has been found that the median lethal orally dose of Schizonepetin was 478 mg/kg in mice and the no observed adverse effect level in rat was 120 mg/kg/day [5,6].

Despite the well documented and widespread pharmacological pharmacokinetic studies of Schizonepetin, little is known about its potential effects on the drug metabolizing enzymes, especially hepatic Cytochrome P450 (CYPs) activity and mRNA expression. CYP is the major family of enzymes involved in metabolism of drugs, toxicants and endogenous compounds, which make up 70%–80% of all phase I xenobiotic metabolizing enzymes. It plays an important role in biotransformation of xenobiotics and endobiotics. The expression of individual CYPs is regulated by both endogenous factors and xenobiotics including drugs and natural compounds. During drug development to avoid undesirable drug-drug/herb interactions that may lead to changes in the rate of drug metabolism and potentially contribute to drug toxicity, it is helpful to preliminarily understand the metabolism of a new chemical entity and its affinity to certain metabolizing enzymes [7–9].

It is of great importance to assess the potential inhibition or induction effects of Schizonepetin on CYPs. Thus, the purpose of this was to investigate the changes of CYP isozymes at activity, mRNA expression levels. In this study, the pharmacokinetic profiles of five substrates drugs: phenacetin, dapsone, chlorzoxazone, omeprazole and metoprolol were selected to evaluate the potential inductive or inhibitory effects on CYP1A2, CYP3A1/2, CYP2E1, CYP2C19, and CYP2D6. In addition, the real-time RT-PCR was used to examine the effects of Schizonepetin on expression of CYP3A1, CYP1A2 and CYP2E1 mRNA in rat.

## 2. Results and Discussion

### 2.1. Effect of Schizonepetin on the Activities of CYP3A1/2 in Rats

**Figure 2:**
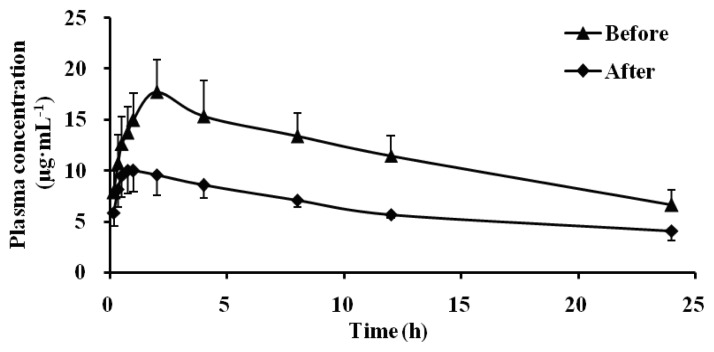
Mean plasma concentration-time curves of dapsone in rats.

The pharmacokinetic profiles of dapsone before and after oral administration of Schizonepetin for seven days were shown in Table 1 and Figure 2. Compared with pre-administration, the *T*_max_ of dapsone was significantly decreased from 2.10 h to 0.85 h, the *C*_max_ was significantly decreased (0.57 times of the control group), the AUC_0–_*_t_* was reduced 37%, and the half-life time was 1.22 times of the control group, the CL increased by 63%. Our results indicated that CYP3A1/2 activity significantly induced by Schizonepetin after multiple oral administrations in rats.

### 2.2. Effect of Schizonepetin on the Activities of CYP1A2 in Rats

**Figure 3:**
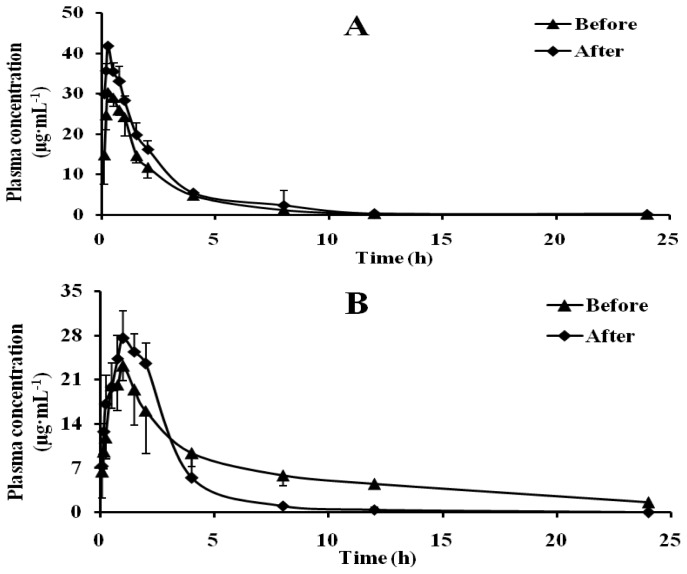
Mean plasma concentration-time curves of Phenacetin (**A**) and Acetaminophen (**B**) in rat plasma.

As shown in Table 2 and Figure 3, compared with pre-administration, the *T*_max_ of phenacetin shortened, *t*_1/2_ changed little, the *C*_max_ and AUC increased, CL decreased. Although the *C*_max_ of metabolite Acetaminophen increased, its *t*_1/2_ significantly decreased, AUC decreased, CL increased. According to the data, it indicated that CYP1A2 activity significantly inhibited by Schizonepetin after multiple oral administrations in rats.

### 2.3. Effect of Schizonepetin on the Activities of CYP2E1 in Rats

**Figure 4:**
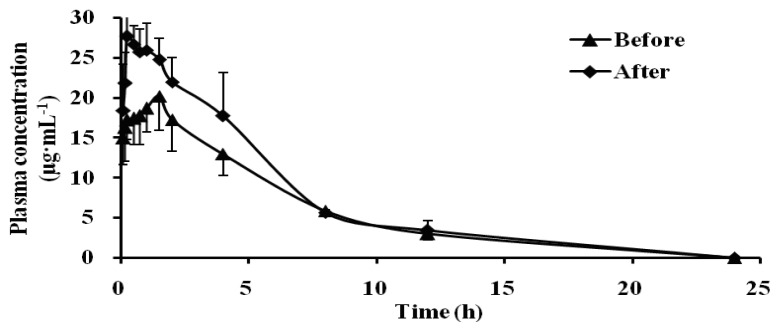
Mean plasma concentration-time curves of Chlorzoxazone in rat plasma.

As shown in Table 3 and Figure 4, compared with pre-administration, the *T*_max_ of Chlorzoxazone became 0.25 h from 1.44 h, the *C*_max_ significantly increased (1.76 times of the control group), the AUC increased to 1.36 times of the control group, the CL significantly decreased. According to the data, it indicated that CYP2E1 activity was significantly inhibited by Schizonepetin after multiple oral administrations in rats.

### 2.4. Effect of Schizonepetin on the Activities of CYP2C19 in Rats

**Figure 5:**
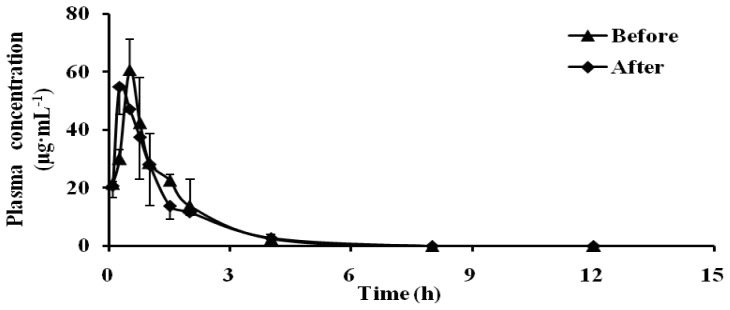
Mean plasma concentration-time curves of Omeprazole in rats.

As shown in Table 4 and Figure 5, compared with pre-administration, the pharmacokinetic parameters of omeprazole showed no significant change. According to the data, it indicated that Schizonepetin has no inductive or inhibitory effect on the activity of CYP2C19 after multiple oral administrations in rats.

### 2.5. Effect of Schizonepetin on the Activities of CYP2D6 in Rats

**Figure 6:**
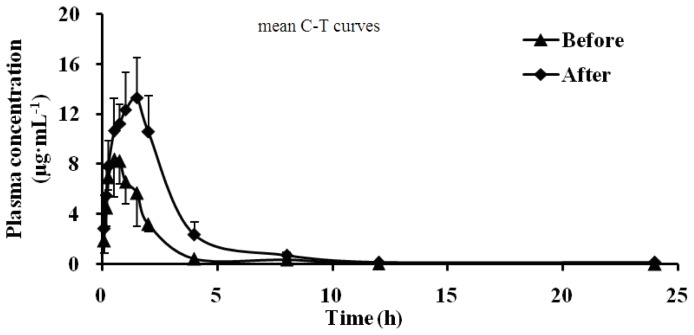
Mean plasma concentration-time curves of Metoprolol in rat plasma.

As shown in Table 5 and Figure 6, compared with pre-administration, the *T*_max_ of metoprolol becomes 1.33 h from 0.5 h, the *C*_max_ significantly increased (1.51 times of the control group), the AUC increased to 2.47 times of the control group, the CL decreased (0.45 times of the control group). According to the data, it indicated that CYP2D6 activity significantly inhibited by Schizonepetin after multiple oral administrations in rats.

### 2.6. Effect of Schizonepetin on the Activities of CYP3A1, CYP1A2 and CYP2E1 mRNA Expression in Rats

After multiple oral administrations, compared with the control group, the average body weight of the Schizonepetin treated rats only increased slightly, with no statistical significance (data not show). These results indicated that Schizonepetin had no significantly effect on the growth rate of rats.

**Figure 7:**
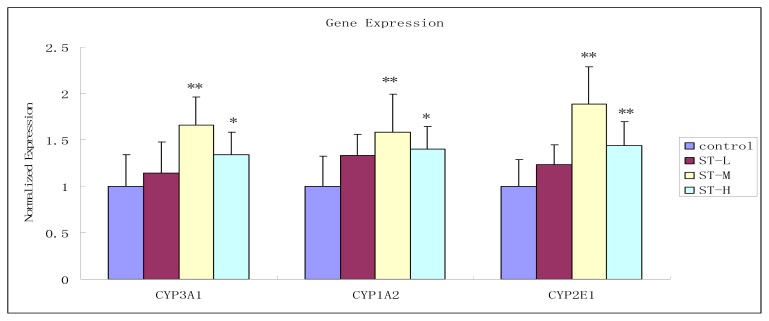
Effect of Schizonepetin on mRNA expression of CYP3A1, CYP1A2 and CYP2E1. Control: control group; ST-L: low group; ST-M: middle group; ST-H: high group; * p < 0.05 *vs*. control; ** *p* < 0.01 *vs*. control.

As shown in Figure 7, compared with the control group, no significant difference was found among the level of CYP3A1, CYP1A2 and CYP2E1 of low dosage Schizonepetin treated group (*p* > 0.05). The level of CYP3A1, CYP1A2 and CYP2E1 in middle dosage Schizonepetin treated group were significantly increased to 1.66, 1.58, 1.88 times of the control group (*p* < 0.01). The level of CYP3A1, CYP1A2 and CYP2E1 in high dosage treated group were 1.34, 1.40, 1.44 times to the control group, and the difference was also significant (*p* < 0.05). However, its relative mRNA levels were lower than the middle dose group. These results indicated that Schizonepetin has inductive effect on the activity of CYP3A1, CYP1A2 and CYP2E1 at middle and high dosage while not at low dosage. The levels of mRNA were lower in the high dose group as compared to the middle dose group which suggests cytochrome P450 enzymes are being induced by Schizonepetin..

### 2.7. Discussion

CYPs, an important factor in drug metabolism, are clinically involved in the drug-drug/herb interactions by CYPs inhibition and/or CYPs induction. Among these CYPs subtypes, CYP1A, 2B, 2C, 2E and 3A, are the main subtypes contribute to the metabolism of drugs in clinics [10,11]. In this study, we mainly focused on the influence of Schizonepetin on the activity and mRNA expression of five major CYP isozymes.

Many methods are available to measure the enzyme activity of CYPs. The probe substrate approach can be used as a general screening tool to characterize CYPs’ inhibition and induction effects for a drug [12]. It can not only detect the activity of a variety of drug-metabolizing enzyme, but also get the inductive or inhibitory effect by the change of the activity of the one enzyme. The cocktail approach has been, in general, proposed as a screening tool for potential *in vivo* drug-drug interactions. As compared with the individual administration of specific probes, cocktail approach was of several distinct advantages, which could provide more inhibition or induction information. However, this method was vigorously debated for some deficiencies which included potential drug-drug interactions among the probes, tolerability and toxicity of some probes, requirements for sensitive and specific assays, and factors other than genetic polymorphism and induction that may alter the pharmacokinetic profiles of the probes under investigation [13,14].

Thus, in our study, a probe substrate approach by HPLC was established and validated to evaluate the inductive or inhibitory effects on CYP3A1/2 CYP1A2, CYP2E1, CYP2C19 and CYP2D6 by pharmacokinetic parameters of five representative substrate drugs dapsone, phenacetin, chlorzoxazone, omeprazole and metoprolol, respectively [15]. This study also detected the concentration of acetaminophen, the major metabolite of phenacetin in rat plasma, which could reflect the changes of CYP1A2 activity more clearly and sensitively. Three cytochrome P450 gene families (those being CYP1, CYP2, and CYP3) appear to be responsible for the majority of drug metabolism. Therefore, Real-time RT-PCR was used to examine CYP3A1, CYP1A2 and CYP2E1 mRNA expression [16].

CYP3A was known as the rate-limiting step in the metabolism and clearance of a large variety of clinical medications, including many pediatric drugs [17]. In the present study, we study the activity of CYP3A by dapsone as probe substrate. According to Figures 2 and 7, it indicated that CYP3A1/2 activity significantly induced by Schizonepetin after multiple oral administrations in rats. The level of mRNA expressions of CYP3A significantly increased at middle and high dosage of Schizonepetin. This was in consistent with the pharmacokinetic results. These indicated that Schizonepetin could induce the CYP3A. As the level of mRNA expressions of CYP3A at high dosage below the middle dosage, it suggested that Cytochrome P450 Enzymes may be induced its own metabolism by Schizonepetin at high dosage. The above results show that when Schizonepetin is used in combination with other drugs which metabolized by the CYP3A1, the potential drug-drug interactions should be pay more attention so as to reduce some adverse reactions or the failure in treatment due to low plasma concentration.

CYP1A2 is involved in the metabolism of several endogenous compounds and some widely used drugs, also it could activate the procarcinogens such as aflatoxin B1, the commonly recognized hepatocarcinogen [18]. CYP2E1 has a unique capacity to activate many xenobiotics to hepatotoxic or carcinogenic products. CYP2E1 is responsible for the metabolism of a large number of low-molecular-weight chemicals, such as aliphatic, aromatic, and halogenated hydrocarbons [19]. Due to its ability to metabolize the compounds, CYP2E1 may be an important determinant factor of humans’ susceptibility to toxicity and carcinogenicity of industrial and environmental chemicals. Therefore, the induction or inhibition on activity of CYP1A2 and CYP2E1 may lead to some undesirable effects. According to our results, both CYP1A2 and CYP2E1 activity could be significantly inhibited by Schizonepetin after multiple oral administrations in rats.

The study results also show that Schizonepetin can induce the mRNA expression of CYP1A2 and CYP2E1 in high dosage (48 mg/kg). It may lead to the accumulation of carcinogenic metabolites *in vivo*, and it is harmful to the human body when it is used at long-term and high-dose. Dosage and course of Schizonepetin for treatment should be cautioned.

In addition, the study also finds that there was no significant difference of the pharmacokinetic parameters of the omeprazole group before and after administration of Schizonepetin. It suggests that Schizonepetin has no inductive or inhibitory effect on the activity of CYP2C19 after multiple oral administrations in rats.

The PK results reflect general level and the PCR data only reflect the gene level. From the PCR data about up-regulation of CYP1A2 and CYP2E1, it is in contrast to the PK results for the same dose level of schizonepetin (24 mg/kg). It indicated that Schizonepetin not only had effects on gene, but also on other ways, which can inhibit CYP1A2 and CYP2E1. The final result is that Schizonepetin had inhibition effects on CYP1A2 and CYP2E1.

## 3. Experimental Section

### 3.1. Experimental Material and Instrument

#### 3.1.1. Apparatus

The HPLC system consisted of a Waters 515 pumps, 717 plus Autosampler, 2487 Dual λ Absorbance Detector and an Empower Pro workstation (Waters Co., Milford, MA, USA), LIBROR AEL-40SM electronic analytical balance (SHIMADZU Co., Kyoto, Japan), Nitrogen Evaporators (HENGAO T & D, Tianjin, China), Anke TGL-16C Table Centrifuge (Shanghai Anting Scientific Instrument Co., Ltd, Shanghai, China), absorbance microplate reader (Molecular device Spectra max plus 384, Sunnyvale, CA, USA), MiniOpticon™ Real-Time PCR Detection System (Bio-Rad Laboratories, Hercules, CA, USA).

#### 3.1.2. Chemicals and Reagents

Schizonepetin was isolated in our laboratory with its purity higher than 99%, which was validated by HPLC and NMR analysis. Tinidazole, omeprazole and metoprolol were purchased from the National Institute for the Control of Pharmaceutical and Biological Products (Beijing, China). Dapsone and phenacetin was purchased from Aladdin reagent Co., Ltd. (Shanghai, China). 4-acetaminophen and Ethylparaben were purchased from Sinopharm Chemical Reagent Co., Ltd. (shanghai, China). Chlorzoxazone was purchased from Research Institute for Liver Diseases (Shanghai, China) Co., Ltd. HPLC grade acetonitrile were obtained from Merck KGaA (Darmstadt, Germany). HPLC grade Methanol was obtained from Jiangsu Hanbon Science & Technology CO., Ltd. (Huai’an, China). Other reagents and chemicals were of analytical grade.

#### 3.1.3. Animals

Male Sprague-Dawley rats (male, 180–220 g) obtained from the laboratory animal center of Zhejiang province were housed under standard environmental conditions (22 ± 2 °C, relative humidity 55% ± 5%, 12-h light-dark cycle) with free access to standard laboratory chow and water *ad libitum*. All experimental procedures were performed in accordance with the Animal Ethics Committee of China Pharmaceutical University.

### 3.2. *In Vivo* Assay

#### 3.2.1. Drug Administration and Sampling

Twenty five male Sprague-Dawley rats were randomly divided into the following groups: dapsone group, phenacetin group, chlorzoxazone group, omeprazole group, metoprolol group. Each group contained five rats. Before Schizonepetin solution was administered, the probe substrates dapsone, phenacetin, chlorzoxazone, omeprazole and metoprolol solutions were administered orally at a dosage of 60, 360, 60, 80, 30 mg·kg^−1^, respectively. Then blood samples (0.2–0.22 mL) were obtained from fossa orbitalis vein according to the heparinized micro-centrifuge tubes at 0 min, 5 min, 10 min, 15 min, 30 min, 45 min, 1 h, 1.5 h, 2 h, 4 h, 8 h, 12 h and 24 h, respectively. From the 7th blood collection, the rats were treated by oral administration of normal saline of the same blood collection volume in order to restore blood capacity quickly. Each rat was orally administered with Schizonepetin (dissolved in 0.5% CMC-Na solution) at a dosage of 24 mg/kg for a consecutive seven days. In the 8th day, the probe substrates were administered as described previously. In addition, blood samples were obtained as before. Blood samples were harvested by centrifuging the blood at 12,000× *g* for 5 min and stored at −20 °C until analysis.

#### 3.2.2. Sample Extraction Procedure

In our study, a conventional liquid-liquid extraction method was used to prepare the plasma sample. After 100 μL of the plasma sample was transferred into a 1.5 mL EP tube, 20 μL internal standard was added, as well as 500 μL of Ethyl Acetate. The resulting solution was thoroughly vortex-mixed for 2 min. After ultrasonic extraction for 5 min, the supernatant was collected after centrifugation at 12,000× *g* for 5 min and evaporated to dryness at 40 °C under a gentle stream of nitrogen. Finally, the residue was reconstituted with 100 μL mobile phase and centrifuged at 12,000× *g* for 10 min; 20 μL aliquot was injected into the HPLC system for analysis.

#### 3.2.3. Chromatographic Methods

Chromatographic separation was achieved on a Megres-C_18_ column (4.6 mm × 150 mm i.d., 5 μm, Jiangsu Hanbon, Huai’an, China) with a pre-column Phenomenex KJ0-4282 (4.0 mm × 3.0 mm). The column temperature was maintained at 40 °C and the flow rate was 1.0 mL/min. The detailed chromatographic conditions for each probe substrates were shown in Table 6.

#### 3.2.4. Statistical Analysis

The pharmacokinetic parameters were calculated by pharmacokinetics program (Version Kinetica 4.4.1 edited by the Thermo Electron Corp, (Philadelphia, PA, USA, 2007). All data were presented as the mean ± SD. *T*-test was employed to determine the difference of pharmacokinetic parameters data between the two groups, for all analyses, *p* < 0.05 was considered statistically significant.

### 3.3. Method of Effects of Schizonepetin on mRNA Expression of Cytochrome P450 Enzymes in Rat

#### 3.3.1. Drug Administration and Sampling

Twelve Male Sprague-Dawley rats were randomly divided into four groups. Three groups were given Schizonepetin (dissolved in 0.5% CMC-Na solution) at doses of 12, 24 and 48 mg/kg by gavage while the other group received 0.5% CMC-Na solution in the same amount, once daily for a consecutive seven days. Twenty-four hours after last dose, rats were killed. The rats were fasted for 12 h with free access to water before the rats were killed. Each liver was removed, and store at −80 °C.

#### 3.3.2. Total RNA Isolation

Total RNA was isolated by TRIzol reagent (Invitrogen, Calsbad, CA, USA) according to the supplier’s instructions. RNA was quantified by optical density measurements at 260 and 280 nm. Integrity was confirmed by running samples on 1% agarose gel.

#### 3.3.3. Synthesis of cDNA

We have used 2 μL RNA in a 20 μL reaction mixture utilizing RevertAid™ M-MuLV RT (Fermentas, Hanover, MD, USA) according to the supplier’s instructions. Resulting reverse transcription products were stored at −70 °C until assay.

#### 3.3.4. Polymerase Chain Reaction

Reactions were performed in a final volume of 24 μL that contained Platinum SYBR Green qPCR SuperMix-UDG 1 12.5 μL, 1 μL cDNA, 1 μL Reverse primer (10 μM), and autoclaved distilled water.

The PCR conditions were: 95 °C for 3 min, followed by 44 cycles of 15 s denaturation at 95 °C and 1 min annealing at 60 °C. The sequences of primers used in this experiment are summarized as follows:

GAPDH:
Forward: 5′-CAAGGTCATCCATGACAACTTTG-3′
Reverse: 5′-GTCCACCACCCTGTTGCTGTAG-3′
CYP1A2:
Forward: 5′-TCAACCTCGTGAAGAGCAGCA-3′
Reverse: 5′-GTCCTGGATACTGTTCTTGTTGAAGTC-3′
CYP2E1:
Forward: 5′-GACCAAAGGCCAGCCTTTTG-3′
Reverse: 5′-GTTATTGTAAAGCTGGATCCAGGGG-3′
CYP3A1:
Forward: 5′-TCTGTGCAGAAGCATCGAGTG-3′
Reverse: 5′-TGGGAGGTGCCTTATTGGG-3′

#### 3.3.5. Statistical Analysis

Data are presented as the mean ± SD. Statistical comparisons were made by one-way ANOVA followed by Tukey’s post hoc test.

## 4. Conclusions

In summary, our results suggest that Schizonepetin has significant induction effects on CYP3A1/2 and inhibition effects on CYP1A2, CYP2E1 or CYP2D6, while it has no effect on CYP2C19. Furthermore, the level of CYP3A1, CYP1A2 and CYP2E1 mRNA expression could be induced by Schizonepetin to different degrees. Thus, co-administrated of some CYP substrate with Schizonepetin may lead some undesirable herb-drug interaction.

## Figures and Tables

**Table 1:** Pharmacokinetics parameters of dapsone in rats (*n* = 5, Mean ± SD).

Parameter/Unit	Dapsone	

	Before	After
*C*_max_/μg·mL^−1^	17.72 ± 3.19	10.13 ± 2.12
*T*_max_/h	2.10 ± 0.22	0.85 ± 0.14
*t*_1/2_/h	15.77 ± 2.98	19.44 ± 5.24
AUC_0–_*_t_*/μg·h·mL^−1^	272.6 ± 48.89	150.5 ± 14.12
AUC_0–∞_/μg·h·mL^−1^	425.3 ± 98.30	268.6 ± 56.34
MRT_0–_*_t_*/h	10.29 ± 0.18	10.19 ± 0.64
MRT_0–∞_/h	23.35 ± 3.91	28.53 ± 7.85
CL/L·h^−1^	32.68 ± 6.95	53.20 ± 10.04
Vz/L	0.73 ± 0.17	1.45 ± 0.32

**Table 2:** Pharmacokinetics parameters of Phenacetin and Acetaminophen in rat plasma (*n* = 5, Mean ± SD).

Parameter/Unit	Phenacetin		Acetaminophen	

	Before	After	Before	After
*C*_max_/μg·mL^−1^	30.94 ± 0.39	41.72 ± 0.38	23.4 ± 2.83	29.90 ± 6.60
*T*_max_/h	0.31 ± 0.13	0.25 ± 0.00	1.13 ± 0.25	1.10 ± 0.22
*t*_1/2_/h	2.32 ± 0.56	2.38 ± 0.57	7.79 ± 1.38	1.67 ± 0.25
AUC_0–_*_t_*/μg·h·mL^−1^	67.48 ± 4.87	90.35 ± 19.21	143.5 ± 32.55	89.45 ± 23.91
AUC_0–∞_/μg·h·mL^−1^	70.43 ± 7.97	91.08 ± 18.99	162.1 ± 39.81	90.68 ± 24.03
MRT_0–_*_t_*/h	2.34 ± 0.36	2.52 ± 0.36	7.33 ± 0.17	2.40 ± 0.26
MRT_0–∞_/h	2.78 ± 0.52	2.72 ± 0.23	10.52 ± 1.16	2.54 ± 0.21
CL/L·h^−1^	1.11 ± 0.16	0.92 ± 0.14	0.49 ± 0.10	0.95 ± 0.25
Vz/L	3.65 ± 0.76	3.25 ± 1.18	5.62 ± 1.31	2.25 ± 0.39

**Table 3:** Pharmacokinetics parameters of Chlorzoxazone in rat plasma (*n* = 5, Mean ± SD).

Parameter/Unit	Chlorzoxazone	

	Before	After
*C*_max_/μg·mL^−1^	20.20 ± 4.30	35.56 ± 6.96
*T*_max_/h	1.44 ± 0.52	0.25 ± 0.00
*t*_1/2_/h	3.32 ± 0.89	3.35 ± 1.13
AUC_0–_*_t_*/μg·h·mL^−1^	120.3 ± 16.75	165.0 ± 40.03
AUC_0–∞_/μg·h·mL^−1^	133.5 ± 26.55	181.8 ± 41.34
MRT_0–_*_t_*/h	4.39 ± 0.50	3.94 ± 0.25
MRT_0–∞_/h	5.58 ± 0.62	5.20 ± 0.59
CL/L·h^−1^	0.11 ± 0.03	0.08 ± 0.03
Vz/L	0.52 ± 0.06	0.42 ± 0.25

**Table 4:** Pharmacokinetics parameters of Omeprazole in rat plasma (*n* = 5, Mean ± SD).

Parameter/Unit	Omeprazole	

	Before	After
*C*_max_/μg·mL^−1^	60.76 ± 10.53	55.69 ± 9.71
*T*_max_/h	0.42 ± 0.14	0.38 ± 0.18
*t*_1/2_/h	0.76 ± 0.25	1.03 ± 0.25
AUC_0–_*_t_*/μg·h·mL^−1^	71.11 ± 20.06	67.13 ± 15.97
AUC_0–∞_/μg·h·mL^−1^	74.02 ± 20.48	71.66 ± 19.43
MRT_0–_*_t_*/h	1.18 ± 0.10	1.14 ± 0.07
MRT_0–∞_/h	1.34 ± 0.16	1.40 ± 0.23
CL/L·h^−1^	0.09 ± 0.02	0.09 ± 0.02
Vz/L	0.10 ± 0.05	0.13 ± 0.01

**Table 5:** Pharmacokinetics parameters of Metoprolol in rat plasma (*n* = 5, Mean ± SD).

Parameter/Unit	Metoprolol	

	Before	After
*C*_max_/μg·mL^−1^	9.47 ± 1.74	14.34 ± 2.57
*T*_max_/h	0.50 ± 0.20	1.33 ± 0.29
*t*_1/2_/h	1.32 ± 0.58	2.91 ± 1.61
AUC_0–_*_t_*/μg·h·mL^−1^	15.89 ± 2.03	37.89 ± 2.86
AUC_0–∞_/μg·h·mL^−1^	16.38 ± 1.95	40.52 ± 5.05
MRT_0–_*_t_*/h	1.75 ± 0.42	3.01 ± 1.09
MRT_0–∞_/h	2.01 ± 0.52	3.71 ± 1.34
CL/L·h^−1^	1.04 ± 0.18	0.47 ± 0.07
Vz/L	2.05 ± 1.05	2.07 ± 1.29

**Table 6:** Chromatographic condition.

Probe substrate	Mobile phase	ë	Internal standard
Dapsone	A (methanol):B (water, 0.02% Triethylamine, pH 3.0) = 80:20 (*v*/*v*)	292 m	tinidazole 0.25 mg·mL^−1^
Phenacetin	A (methanol):B (water), gradient elution, 0–4 min (35% A, 65% B), 5–20 min (45% A, 55% B), 21–23 min (35% A, 65% B)	245 m	4-acetaminophen 0.2 mg·mL^−1^
Chlorzoxazone	methanol:water = 48:52 (*v*/*v*)	278 m	omeprazole 0.25 mg·mL^−1^
Omeprazole	A (methanol):B (water), gradient elution, 0–7 min (52% A, 48% B), 8–12 min (40% A, 60% B), 13–17 min (52% A, 48% B)	300 m	coumarin 0.1 mg·mL^−1^
Metoprolol	A (methanol):B (water, 0.02% Triethylamine, pH 3.5) = 40:60 (*v*/*v*)	225 m	phenacetin 0.25 mg·mL^−1^
